# Histone acetylation risk model predicts prognosis and guides therapy selection in glioblastoma: implications for chemotherapy and anti-CTLA-4 immunotherapy

**DOI:** 10.1186/s12865-024-00639-7

**Published:** 2024-07-27

**Authors:** Xingyi Jin, Zhigang Qin, Hang Zhao

**Affiliations:** Neurosurgery Department, China-Japan Union Hospital of Jilin University, Changchun, Jilin China

**Keywords:** Glioblastoma, Histone acetylation, Personalized treatment, CTLA4, Topotecan

## Abstract

**Background:**

Glioblastoma is characterized by high aggressiveness, frequent recurrence, and poor prognosis. Histone acetylation-associated genes have been implicated in its occurrence and development, yet their predictive ability in glioblastoma prognosis remains unclear.

**Results:**

This study constructs a histone acetylation risk model using Cox and LASSO regression analyses to evaluate glioblastoma prognosis. We assessed the model’s prognostic ability with univariate and multivariate Cox regression analyses. Additionally, immune infiltration was evaluated using ESTIMATE and TIMER algorithms, and the SubMAP algorithm was utilized to predict responses to CTLA4 inhibitor. Multiple drug databases were applied to assess drug sensitivity in high- and low-risk groups. Our results indicate that the histone acetylation risk model is independent and reliable in predicting prognosis.

**Conclusions:**

Low-risk patients showed higher immune activity and longer overall survival, suggesting anti-CTLA4 immunotherapy suitability, while high-risk patients might benefit more from chemotherapy. This model could guide personalized therapy selection for glioblastoma patients.

**Supplementary Information:**

The online version contains supplementary material available at 10.1186/s12865-024-00639-7.

## Background

Glioblastoma is the most common and aggressive primary malignant brain tumor, accounting for a significant percentage of all such tumors [1]. According to the latest World Health Organization (WHO) classification of brain tumors, glioblastoma is characterized by its highly aggressive nature and poor prognosis [2]. Despite advances in medical research, the prognosis for glioblastoma remains grim, with only about 30% of patients surviving for one year and fewer than 5% surviving for five years [3]. This dire situation underscores the urgent need for improved prognostic models and therapeutic strategies.

Histone acetylation, a critical epigenetic modification, has been increasingly recognized for its role in gene regulation and cancer progression [4]. Previous studies have established a link between histone acetylation-associated genes and the development, prognosis, and recurrence of glioblastoma [5, 6]. However, the predictive capacity of histone acetylation regulators in glioblastoma has not been fully elucidated.

In this study, we aim to develop a novel histone acetylation risk model to better predict the prognosis of glioblastoma patients. Using univariate Cox and LASSO regression analyses, we identified key histone acetylation regulators that contribute to patient outcomes. We then evaluated the prognostic performance of this model through univariate and multivariate Cox regression analyses. Additionally, we assessed the immune landscape of glioblastoma patients’ tumor microenvironments using ESTIMATE and TIMER algorithms. Understanding the immune contexture is crucial, as it influences tumor behavior and patient response to therapies. To further explore therapeutic implications, we predicted the response of high- and low-risk patient groups to immune checkpoint inhibitors targeting PD-1 and CTLA4 using the SubMAP algorithm. We also examined drug sensitivity across these groups using data from multiple drug datasets. Our findings demonstrate that the histone acetylation risk model is robust, independent, and reliable, offering accurate prognostic predictions. The model suggests that high-risk patients may benefit from chemotherapy, while low-risk patients might be more suitable candidates for anti-CTLA4 immunotherapy. Collectively, our research provides valuable insights into glioblastoma prognosis and potential therapeutic strategies, guiding personalized treatment approaches.

## Methods

### Data acquisition

To construct our training set, we meticulously collected glioblastoma data from the TCGA database. We excluded samples lacking complete survival data to ensure the integrity and reliability of our analysis. For validation purposes, we incorporated additional datasets from the CGGA and GlioVis databases. These databases are recognized for their extensive glioma research data, and we adhered to strict criteria for data completeness and reliability to maintain consistency [7, 8].

### Single-cell data processing and analysis

We acquired single-cell RNA sequencing data for glioblastoma from the GEO database, specifically dataset GSE182109. The GEO database is a valuable repository for high-throughput gene expression data, providing crucial insights into cellular functions. To preprocess the data, we excluded genes with zero expression across all samples, as their lack of variability would not contribute to meaningful analysis. The gene expression matrix was normalized using the “SCTransform” function from the Seurat R package, a method known for its efficacy in single-cell data normalization.

Subsequently, we performed Principal Component Analysis (PCA) and Uniform Manifold Approximation and Projection (UMAP) to reduce dimensionality and visualize the data structure. Cell classification was conducted using the FindNeighbors and FindClusters functions, which are integral to the Seurat workflow for identifying cell populations. Doublets, which can confound single-cell data analysis, were filtered out using the DoubletFinder R package.

Further quality control steps included the removal of cells with more than 15% mitochondrial gene content or fewer than 500 detected genes, as these could indicate low-quality or dying cells. After stringent quality control, approximately 48,827 cells remained. These cells underwent cell-type annotation using the Celltypist package in Python, which leverages reference datasets to accurately assign cell identities, enhancing the biological relevance of our findings.

### Establishment of HA-score

To explore the role of histone acetylation in glioblastoma, we performed a differential gene expression analysis comparing glioblastoma and normal tissues using data from the GTEx-TCGA dataset. The GTEx database, known for its extensive collection of normal tissue gene expression data, combined with the cancer-specific data from TCGA, provided a comprehensive basis for our analysis.

We identified differentially expressed genes (DEGs) and visualized these results using a heatmap, which allowed us to clearly distinguish patterns of gene expression between glioblastoma and normal tissues. To understand the interactions and relationships between these genes, we conducted a correlation analysis using the igraph package. This package is widely recognized for its robust tools for network analysis and visualization, which facilitated the identification of key gene interactions and potential regulatory networks influenced by histone acetylation.

To quantify histone acetylation levels, we developed a histone acetylation score (HA-score) based on the expression levels of differentially expressed histone acetylation regulators. For bulk tissue data, the HA-score was calculated using the single-sample Gene Set Enrichment Analysis (ssGSEA) algorithm, which allows for the assessment of specific gene set activity within a sample. For single-cell RNA sequencing data, we utilized the Ucell algorithm, an efficient method for calculating enrichment scores at the single-cell level [9, 10].

This multifaceted approach enabled us to dissect the complex role of histone acetylation in glioblastoma, providing insights into how these epigenetic modifications may contribute to tumorigenesis and progression.

### Development and validation of HA-model

To pinpoint histone acetylation regulators predictive of glioblastoma outcomes, we conducted a univariate Cox regression analysis on the differentially expressed histone acetylation regulators in our training set. This analysis identified 13 regulators significantly associated with glioblastoma prognosis, highlighting their potential as biomarkers.

For the prognostic analysis, we focused on overall survival (OS) metrics. We applied lasso regression to refine the selection of histone acetylation regulators and constructed a predictive model. Lasso regression is particularly useful for enhancing model performance by penalizing overfitting, thereby selecting only the most informative variables.

The mathematical formula for calculating the risk score is as follows:

$$\varvec{r}\varvec{i}\varvec{s}\varvec{k}\varvec{s}\varvec{c}\varvec{o}\varvec{r}\varvec{e}={\sum }_{\varvec{i}=1}^{\varvec{n}}\left({\varvec{\beta }}_{\varvec{i}}{\varvec{E}\varvec{x}\varvec{p}}_{\varvec{i}}\right)$$

where 𝑛 represents the number of selected histone acetylation regulators, Exp denotes the gene expression levels, and 𝛽 is the multi-Cox coefficient. This risk score allows for the stratification of patients into distinct risk subgroups based on their calculated scores.

To validate the robustness and generalizability of our risk model, we utilized external datasets. The validity of the risk stratification was further confirmed through Kaplan–Meier (KM) survival analysis, conducted using R v4.2. This analysis demonstrated that the differences in survival outcomes between the high-risk and low-risk subgroups were statistically significant (*P* < 0.05), underscoring the model’s predictive power.

### Assessing risk model reliability and generating nomogram

The prognosis analysis aimed to compare the predictive power of the histone acetylation model against common clinical characteristics such as age, gender, and tumor grades. Using forest plots, we displayed the *P*-values and hazard ratios (HR) to illustrate the relative impact of each factor.

To further assess OS at three specific time points, we created a nomogram using the rms R package. This nomogram incorporated the HA model along with selected demographic and clinical characteristics to provide a visual tool for predicting patient outcomes.

For the validation of our HA-model’s reliability, we integrated it with demographic and clinical factors through multivariable Cox regression analyses. This integration enabled the development of a comprehensive nomogram, projecting 1-, 3-, and 5-year survival probabilities for glioblastoma patients. To ensure the predictive accuracy of the nomogram, we utilized calibration plots, which compare predicted and observed survival probabilities, and Area Under the Curve (AUC) curves to evaluate the model’s performance. These combined methods confirm the HA model’s robustness and its utility in providing accurate prognostic predictions for glioblastoma patients, making it a valuable tool in clinical settings.

### Analysis of immune infiltration

To elucidate critical pathways involved in glioblastoma, we applied the ssGSEA algorithm, implemented through the gsva R package. This analysis enabled the calculation of 20 key pathways, providing insights into the biological processes underlying glioblastoma pathogenesis [11].

Furthermore, to identify the various cell types within the tumor microenvironment (TME), we used CIBERSORT. This method allowed us to deconvolute the complex cell populations present in the TME, highlighting the immune and stromal components [12].

In addition to cell identification, we quantified the stromal score, immunological score, and tumor purity using the ESTIMATE algorithm. These metrics are crucial for understanding the composition and potential immune evasion strategies of the tumor, offering a comprehensive view of the TME [13].

### Estimation of drug target

To identify therapeutic targets for high-risk glioblastoma patients, we acquired comprehensive data on 6,125 compounds from the Drug Repurposing Hub. After eliminating duplicates, we identified 2,249 unique drug targets [14].

To isolate genes with therapeutic potential, we first conducted a Spearman correlation analysis. This analysis correlated the expression levels of targetable genes with the risk scores of glioblastoma patients. Genes exhibiting a correlation coefficient greater than 0.3 with a *P*-value less than 0.05 were considered candidate drug targets associated with poor prognosis. This threshold ensures that identified genes are significantly correlated with high-risk profiles. We then calculated risk scores for brain cell lines from the Cancer Cell Line Encyclopedia project and performed a correlation analysis between the CERES score and the risk score using these cell lines. The CERES score estimates gene dependency, accounting for the effects of copy-number variations, where lower CERES scores indicate higher dependency of cancer cell lines on a given gene [15]. Genes with a correlation coefficient below − 0.3 and a *P*-value less than 0.05 were categorized as drug targets associated with poor prognosis dependence. This analysis identifies genes that glioblastoma cells are highly dependent on, thus serving as critical points for therapeutic intervention.

Therapeutic drug targets for high-risk glioblastoma were those identified through both the Spearman correlation analysis and the CERES score correlation analysis. This dual approach ensures that the selected targets are both correlated with high-risk patient profiles and essential for glioblastoma cell survival, making them promising candidates for drug repurposing efforts.

### Chemotherapeutic response prediction

To predict chemotherapeutic responses, we leveraged two extensive pharmacogenomic datasets: CTRP (Cancer Therapeutics Response Portal) and PRISM. These datasets provide comprehensive drug screening and molecular data across numerous cancer cell lines, enabling precise prediction of drug responses in clinical samples. We conducted differential expression analyses to compare bulk samples with cell lines, as well as within the samples and cell lines themselves. This step was crucial for identifying expression patterns that could inform drug response predictions.

This study utilized the ridge regression model from the pRRophetic package, chosen for its proven reliability across multiple studies. The model was employed to forecast drug responses for clinical samples [16, 17]. Training of the predictive model involved using expression profiles and drug response data specifically from solid Cancer Cell Lines, while excluding those derived from hematopoietic and lymphoid tissues to ensure specificity. This approach aimed to enhance the accuracy and applicability of drug response predictions in glioblastoma. The performance of the model was validated through a default 10-fold cross-validation process. This robust validation technique facilitated accurate estimation of drug responses for clinical samples based on their refined expression profiles.

This comprehensive approach integrates cutting-edge pharmacogenomic data with advanced machine learning techniques to enhance the precision of chemotherapeutic response predictions, potentially guiding more effective treatment strategies for glioblastoma patients.

### Connectivity map analysis

To further identify potential therapeutic agents for glioblastoma, we utilized a Connectivity Map (CMap) analysis. This approach investigates the therapeutic potential of candidate compounds by comparing gene expression profiles from glioblastoma tumor samples with those from normal tissues [18]. We started with a comparative analysis of gene expression between glioblastoma tumors and normal tissues. From this analysis, we selected the top 300 genes with the most significant fold changes, evenly split into 150 up-regulated and 150 down-regulated genes. These genes were then submitted to the CMap platform for further investigation. The gene expression signatures employed by CMap are sourced from both CMap version 1 and the Library of Integrated Network-Based Cellular Signatures database. This comprehensive resource features a collection of 2,429 compounds that have been tested across various cell lines, thus providing a robust foundation for identifying compounds that may have therapeutic benefits for glioblastoma patients.

The CMap analysis yielded a distinct connectivity score for each compound, calibrated on a standardized scale ranging from − 100 to 100. A negative connectivity score indicates a gene expression pattern that opposes the disease-specific expression pattern observed in glioblastoma. Such a pattern suggests that the respective compound may have therapeutic potential by counteracting the gene expression changes associated with the disease.

By leveraging this extensive dataset and analytical approach, we identified compounds that could potentially be repurposed as therapeutic agents for glioblastoma, based on their ability to reverse disease-related gene expression signatures.

### Clinical sample collection and patient stratification

Human specimens were collected from a group of 20 glioblastoma patients undergoing surgical procedures at China-Japan Union Hospital. Histopathological examinations were conducted on all samples, which were stained with Hematoxylin and Eosin (HE) following standard protocols. To ensure accuracy and reliability, diagnostic assessments were independently performed by two pathologists.

Total RNA was isolated using the Trizol method (Invitrogen), a highly regarded technique for RNA extraction. For quantitative real-time PCR (qRT-PCR), we employed the One-Step qPCR Kit (Invitrogen) and the CFX Connect™ Real-Time System (BIO-RAD), strictly adhering to the manufacturers’ instructions. Data analysis utilized the 2^−ΔΔCq^ method, normalizing gene expression levels to GAPDH, which is essential for ensuring consistency and comparability across different samples.

Patients were then stratified into low-risk and high-risk groups based on normalized gene expression levels. This stratification was guided by a threshold derived from the HA-model’s equation, improving the accuracy of patient outcome predictions. Such a stratified approach allows for a more personalized assessment of prognosis and potential therapeutic responses in glioblastoma patients.

### Histological evaluation

To prepare glioma tissue sections for immunohistochemistry (IHC), the following steps were conducted: Tissue sections were deparaffinized and then rehydrated using a series of gradient ethanol solutions. This process is essential to remove paraffin wax and rehydrate the tissues, making them suitable for antibody binding. The sections underwent heat-induced epitope retrieval in citrate buffer at 100 °C for 1 h. This step is crucial for unmasking antigenic sites, thereby enhancing antibody binding. The tissue slices were sequentially incubated with primary antibodies, followed by HRP-conjugated secondary antibodies. This incubation allows for specific binding of antibodies to their target antigens within the tissue. The DAB Peroxidase Substrate Kit was utilized to visualize the antigen-antibody complexes. The DAB substrate reacts with the HRP enzyme, producing a brown color that indicates the presence of the target antigen. The IHC images were captured using a microscope, allowing for detailed examination and analysis of the stained tissue sections. Immunohistochemistry was performed using the following antibodies: CD3 (ab16669, Abcam), CD8 (ab82749, Abcam), CD163 (ab79056, Abcam), and FOXP3 (ab20034, Abcam).

## Results

### Potential role of histone acetylation regulators in glioblastoma

**Fig. 1:**
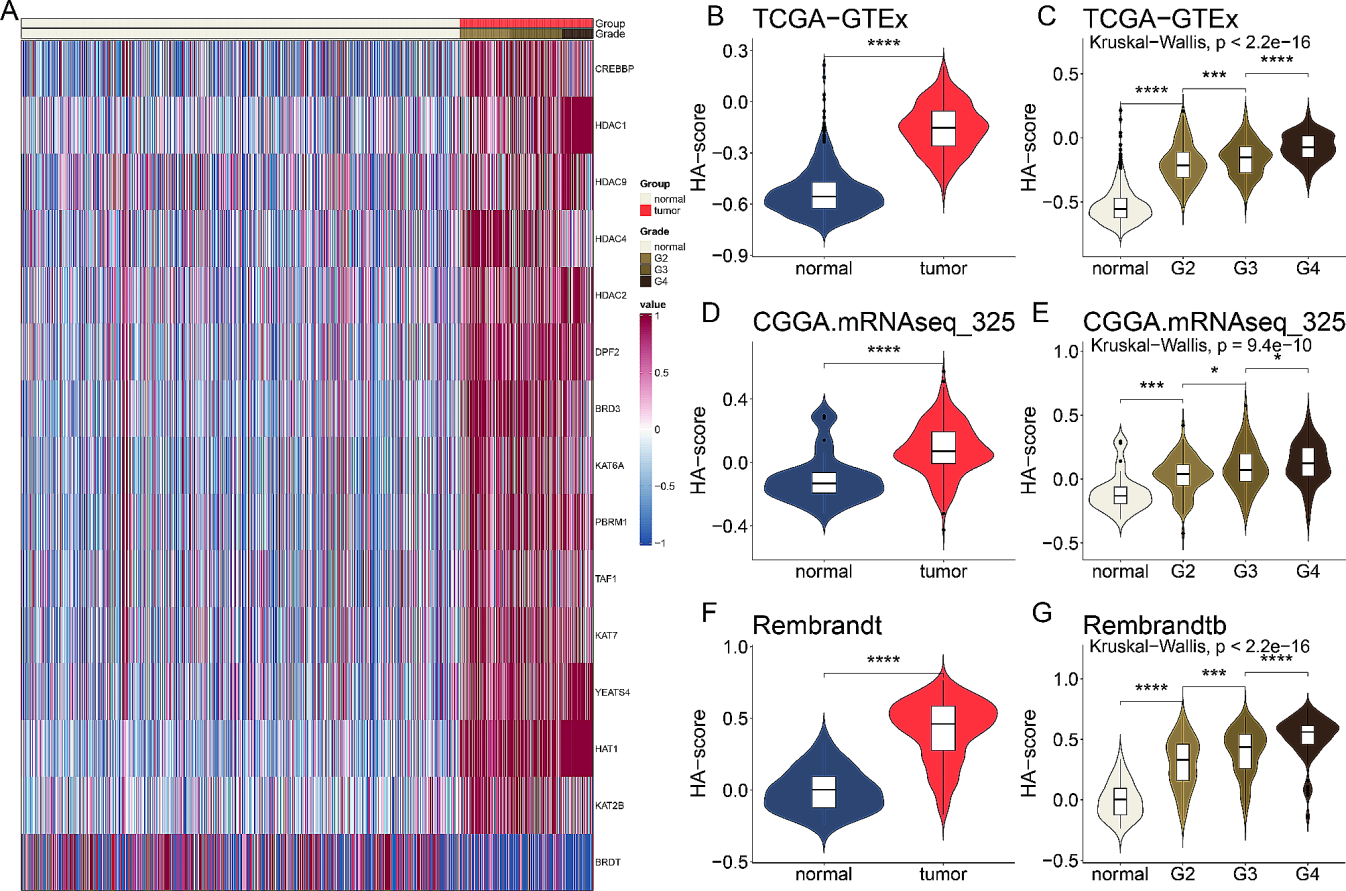
Potential role of histone acetylation regulators in glioblastoma. (**A**) Heatmap displaying the expression profiles of 15 differential histone acetylation regulators in glioblastoma patients compared to normal tissues. (**B**, **D**, **F**) Violin plots comparing the histone acetylation score (HA-score) between normal and glioblastoma tissues across three datasets (TCGA-GTEx, CGGA.mRNAseq_325, and Rembrandt). (**C**, **E**, **G**) Violin plots depicting the HA-score across different clinical stages of glioblastoma in the same datasets, showing a significant correlation between higher HA-scores and advanced clinical stages

Among the 36 histone acetylation regulators, 15 genes exhibited abnormal expression in glioblastoma patients compared to normal tissues, signifying significant biological process variations between these groups. The heatmap displayed the distribution of these 15 differential genes, revealing that one gene was downregulated and 14 genes were significantly upregulated in glioblastoma patients relative to normal cases (Fig. 1A). To systematically explore the relationships among these differential genes, they were categorized into two clusters, and a correlation network was constructed (Figure S1A). We identified strong associations among these genes, such as the synergistic relationship between *HDAC4* and *BRD3* in cluster A and the antagonistic relationship between *HDAC4* and *HAT1* between cluster A and B.

To elucidate the interaction of histone acetylation with glioblastoma, we calculated the histone acetylation score (HA-score) for each glioblastoma patient using the ssGSEA algorithm based on the differential histone acetylation regulators. The HA-score was significantly higher in glioblastoma patients compared to normal individuals in the TCGA-GTEx dataset (Fig. 1B). The HA-score also varied significantly across different clinical stages (normal, G2, G3, G4) in the TCGA-GTEx dataset, showing an increasing trend with advanced stages (Fig. 1C). The elevated HA-score in glioblastoma patients compared to normal individuals was validated in the CGGA.mRNAseq_325 and Rembrandt datasets (Fig. 1D, F). Similarly, the HA-score showed significant variation across clinical stages in the CGGA.mRNAseq_325 and Rembrandt datasets (Fig. 1E, G).

Given the involvement of TME in tumor formation, we assessed the relationship between the HA-score and immune infiltration (Figure S1B). There was a strong positive correlation between the HA-score and M2 macrophages, and a negative correlation with CD8^+^ T cells (Figure S1C, D), indicating that the HA-score may antagonistically influence the hot TME in glioblastoma.

### Assessment of HA-score using the single-cell

**Fig. 2:**
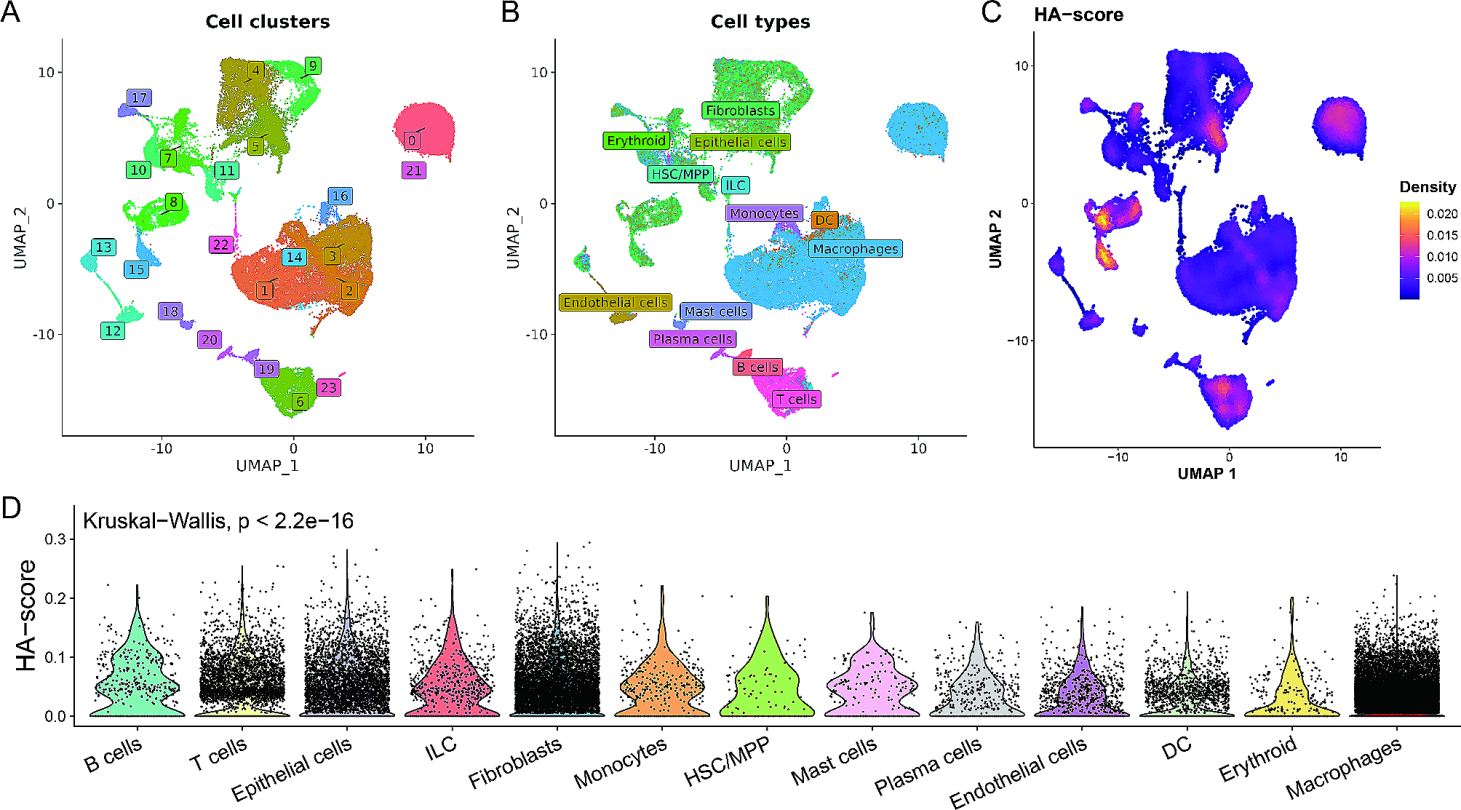
Assessment of HA-score using the single-cell. (**A**) UMAP plot showing the distribution of 48,827 cells into 24 clusters after quality control procedures. (**B**) UMAP plot with annotated cell types using the celltypist algorithm. (**C**) UMAP plot displaying the HA-scores calculated using the Ucell algorithm. (**D**) Violin plots depicting the HA-scores across various immune cell types in glioblastoma samples, showing a significant correlation between HA-scores and immune cell infiltration

To further explore the relationship between the HA-score and the TME at the single-cell level, we analyzed 48,827 cells following stringent quality control procedures. The cells were categorized into 24 distinct clusters using standard clustering algorithms (Fig. 2A). These clusters were annotated using the celltypist algorithm (Fig. 2B), which leverages reference datasets to accurately assign cell identities, providing a detailed map of the cell populations present in the glioblastoma samples. HA-scores were calculated for each cell using the Ucell algorithm (Fig. 2C), an efficient method for assessing histone acetylation status across different cell types. Representative markers for each cell type were labeled, distinguishing each cell type and elucidating their functional roles within the TME (Figure S2A). The top differentially expressed genes were highlighted, providing insights into the cellular heterogeneity and functional diversity within the TME (Figure S2B). Our analysis revealed a significant correlation between HA-scores and immune cell infiltration in glioblastoma samples (Fig. 2D), suggesting that histone acetylation may influence immune responses within the TME and potentially impact tumor progression and patient outcomes. This comprehensive single-cell analysis underscores the interplay between histone acetylation and the immune microenvironment in glioblastoma, highlighting potential therapeutic targets and biomarkers for patient stratification.

### Development of a histone acetylation risk model

**Fig. 3:**
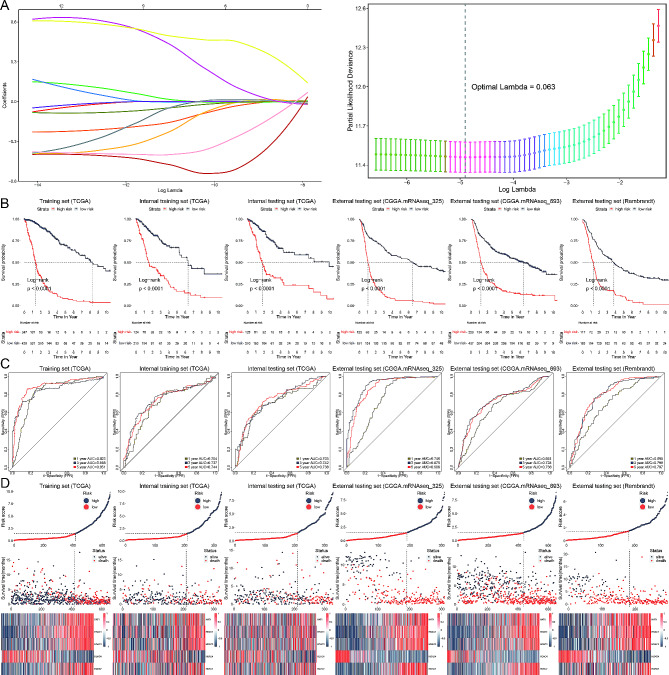
Development of a histone acetylation risk model. (**A**) LASSO regression analysis identifying 13 significant histone acetylation regulators. The plot shows the coefficient paths for these genes as a function of the regularization parameter (Lambda). The right plot determines the optimal Lambda value through cross-validation. (**B**) Kaplan-Meier survival curves for glioblastoma patients stratified into high-risk and low-risk groups based on the histone acetylation risk model (HA-model) in various datasets. Log-rank tests indicate significant survival differences between the groups (*p* < 0.0001). (**C**) Time-dependent ROC curves evaluating the predictive performance of the HA-model at 1, 3, and 5 years in the same datasets. The AUC values exceed 0.5 across all time points, indicating good model performance. (**D**) Risk score distribution and survival status of glioblastoma patients in different datasets, along with heatmaps displaying the expression profiles of the five critical histone acetylation regulators identified by Cox regression analysis. The patients are clearly stratified into high-risk and low-risk groups, with significant survival differences

We used LASSO regression to identify significantly prognostic genes, leading to the selection of 13 histone acetylation regulators (*DFF2*, *HDAC2*, *TAF1*, *PBRM1*, *KAT7*, *KAT6A*, *KAT2B*, *YEATS4*, *CREBBP*, *BRD3*, *HAT1*, *HDAC1*, *HDAC4*) for further analysis (Fig. 3A).

To enhance the model’s reliability, we randomly divided the training set into equal halves for internal training and testing, subsequently assessing it across three external cohorts. Utilizing Cox regression analysis, we identified five critical genes to develop the histone acetylation risk model (HA-model). The formula is shown below:

$$\begin{array}{l}riskscore = HDAC1 \times 0.089 + HDAC3 \times 0.266\\- HDAC4 \times 0.581 + HDAC7 \times 0.377 + HAT1 \times 0.395\end{array}$$

Our model successfully stratified glioblastoma patients into two risk subgroups with significantly different survival probabilities (Fig. 3B). The model’s predictive capability was evaluated using time-dependent ROC analysis at 1, 3, and 5 years, all of which produced AUC values exceeding 0.5, indicating excellent predictive performance (Fig. 3C). Furthermore, the heatmap presents the expression profiles of the five histone acetylation regulators (Fig. 3D).

### Clinical evaluation of the HA-model

**Fig. 4:**
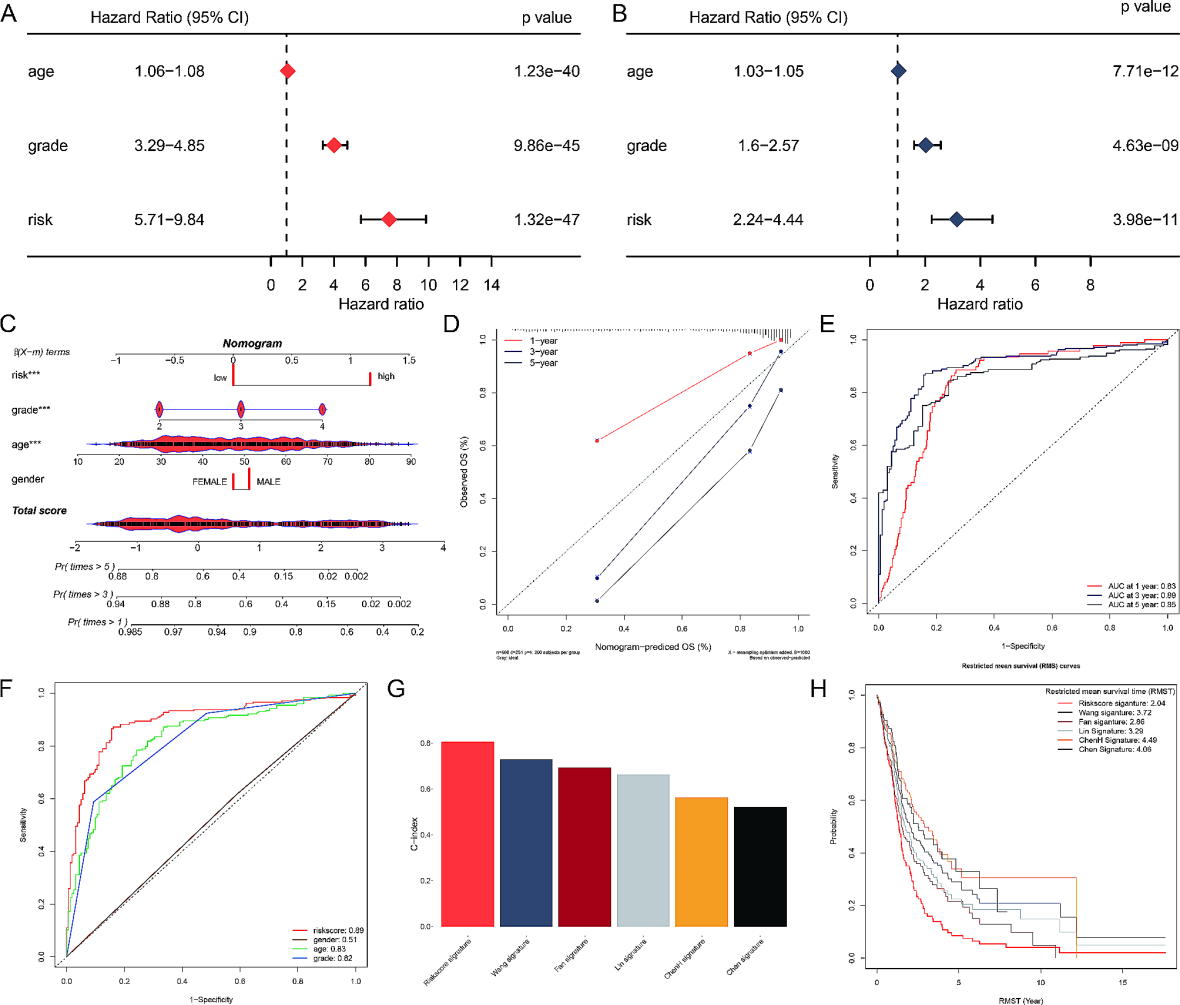
Clinical evaluation of the HA-model. (**A**) Univariate Cox analysis using TCGA data demonstrating the association of age, grade, and risk with glioblastoma prognosis. (**B**) Multivariate Cox analysis using TCGA data confirming the independent prognostic significance of age, grade, and risk for glioblastoma. (**C**) Nomogram integrating the HA-model and standard clinical features (age, grade, gender) to estimate 1-, 3-, and 5-year survival probabilities for glioblastoma patients. (**D**) Calibration plot for the nomogram, illustrating the agreement between predicted and observed 1-, 3-, and 5-year survival probabilities. (**E**) Time-dependent ROC curves evaluating the predictive performance of the HA-model for 1-year (AUC = 0.83), 3-year (AUC = 0.89), and 5-year (AUC = 0.85) survival predictions. (**F**) Comparison of the HA-model’s predictive performance (AUC = 0.89) with other clinical factors including age (AUC = 0.83), sex (AUC = 0.51), and grade (AUC = 0.82), demonstrating superior performance of the HA-model. (**G**) Bar chart comparing the C-index of the HA-model with five existing glioblastoma risk models, highlighting the HA-model’s superior predictive accuracy. (**H**) Restricted mean survival time (RMST) analysis comparing the HA-model with the five existing glioblastoma risk models, showing significant improvement in survival prediction by the HA-model

To adapt the HA-model for clinical application, we assessed its prognostic value using univariate and multivariate Cox analyses with TCGA data (*p* < 0.05) (Fig. 4A, B). These analyses confirmed the model’s reliability in predicting glioblastoma outcomes. We created a nomogram to estimate survival probabilities at 1, 3, and 5 years, integrating both the HA-model and standard clinical features (Fig. 4C). Calibration analysis showed that the nomogram accurately reflected actual survival times (Fig. 4D). The model’s performance was further validated using time-dependent ROC analysis, which yielded survival AUCs of 0.83 for 1-year, 0.89 for 3-year, and 0.85 for 5-year predictions (Fig. 4E). The HA-model’s AUC of 0.89 significantly outperformed other factors such as age (AUC = 0.83), sex (AUC = 0.51), and clinical grade (AUC = 0.82) (Fig. 4F).

Furthermore, we compared our novel model with five existing glioblastoma risk models from the literature [19–23]. Our model demonstrated significant advantages in both C-index and restricted mean survival time (RMST) analysis (Fig. 4G, H), highlighting its superior predictive power and potential for clinical use in stratifying glioblastoma patients and guiding therapeutic decisions.

### Significance of HA-model to clinical indexes and functional variations

A heatmap was generated to illustrate the distribution patterns of five key histone acetylation regulators alongside various clinicopathological factors (Figure S3A). Analysis revealed that the HA-model effectively predicts age, OS status, and clinicopathological grade (Figure S3B). Correlation analysis showed that four genes had positive correlations with the risk score, while one gene had a negative correlation (Figure S3C).

GSEA was performed to explore functional differences between high-risk and low-risk subgroups. Results indicated that the high-risk subgroup had significant enrichment in pathways related to leukocyte-mediated immunity, B cell-mediated immunity, humoral immune response, and Epstein-Barr virus infection. Conversely, pathways such as glucose import, neurotransmitter levels, and AMPK signaling were inhibited in this subgroup (Figures S3D, E).

PCA was conducted on the entire gene set (Figure S3F), on histone acetylation regulators (Figure S3G), and specifically on the five selected histone acetylation regulators from the HA-model (Figure S3H). The expression patterns of these five histone acetylation regulators successfully distinguished between the high-risk and low-risk subgroups.

This thorough investigation demonstrates the HA-model’s robustness in predicting clinical outcomes and highlights the significant biological distinctions between the identified subgroups. These insights are instrumental for developing targeted therapies and improving patient stratification in glioblastoma.

### HA-model correlates immune infiltration and predicts anti-CTLA4 immunotherapy

**Fig. 5:**
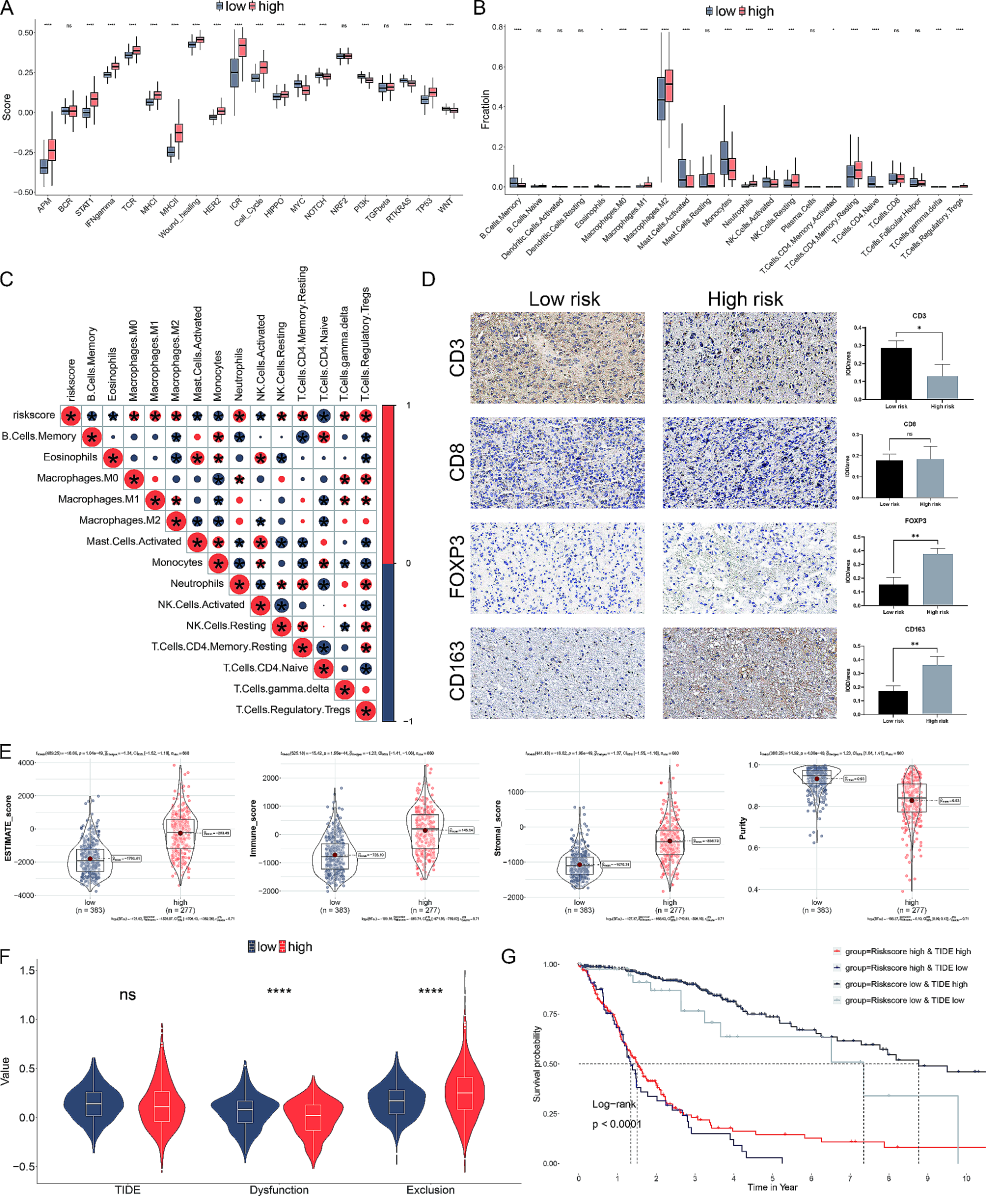
HA-model correlates immune infiltration and predicts anti-CTLA4 immunotherapy. (**A**) Box plots showing the scores of 22 immune cell types in low-risk and high-risk subgroups calculated using CIBERSORT. (**B**) Box plots displaying the scores of 20 associated pathways validated using ssGSEA in the two subgroups. (**C**) Correlation matrix between the risk score and the proportion of various immune cells. (**D**) Immunohistochemical staining for CD3, CD8, FOXP3, and CD163 markers in low-risk and high-risk glioma samples. (**E**) Violin plots showing the results of the ESTIMATE algorithm to evaluate the stromal score, immune score, and tumor purity in different risk subtypes. (**F**) Violin plots illustrating tumor dysfunction and exclusion scores. (**G**) Kaplan-Meier survival curves stratified by risk score and TIDE, demonstrating that patients with higher TIDE and low-risk scores have the most favorable outcomes

To analyze the immune landscape of glioblastoma subgroups, we used CIBERSORT to determine the proportions of 22 immune cell types and ssGSEA to validate the activity of 20 associated pathways (Fig. 5A, B). Low-risk subtypes showed higher levels of specific TME cells, such as activated mast cells, monocytes, B cells, and T cells, but lower infiltration of M2 macrophages and regulatory T cells (Tregs). A notable correlation was observed between the risk score and the proportions of various immune cells (Fig. 5C). Immune cell markers confirmed that high-risk patients had more Tregs and tumor-associated macrophages, but fewer T cells (Fig. 5D).

Using the ESTIMATE algorithm, we assessed stromal scores, immune scores, and tumor purity. Higher risk scores correlated with significantly elevated stromal and immune scores, but lower tumor purity, suggesting potential challenges in immunotherapy effectiveness for these patients (Fig. 5E). Tumor dysfunction and exclusion were more common in the lower risk score subgroup, whereas TIDE scores did not show significant differences (Fig. 5F). Patients with higher TIDE scores and low-risk scores had the most favorable outcomes (Fig. 5G).

**Fig. 6:**
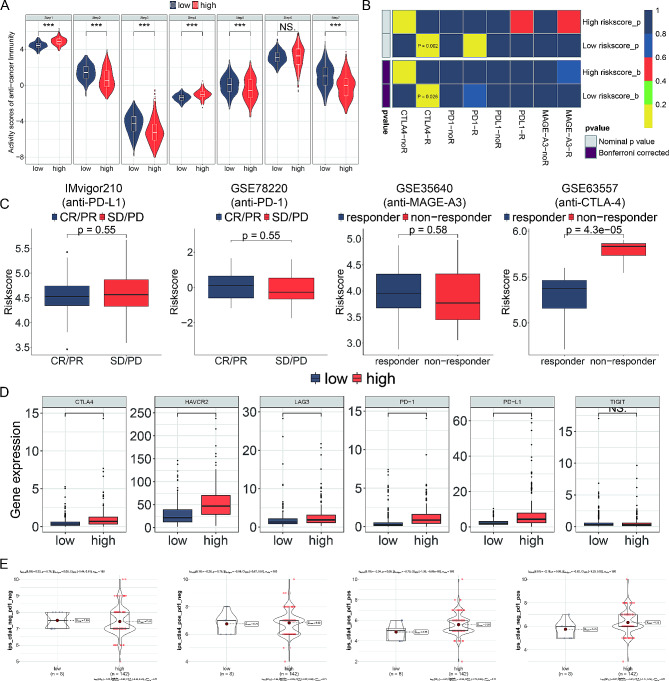
Immune landscape of histone acetylation risk model. (**A**) Pathway activities between the two risk subgroups. (**B**) Differential immune infiltration of 22 immune cell fractions between the two risk subgroups. (**C**) The correlation of 22 immune cell types with the risk score. (**D**) Representative IHC images of immune cell markers between the two risk subgroups. (**E**) Correlation of risk score with the tumor microenvironment. (**F**) TIDE, T cell dysfunction and exclusion between the two risk subgroups. (**G**) Survival analysis of patients with different combinations of risk scores and TIDE in TCGA cohort. (**H**) Correlation of risk score with the intratumor heterogeneity, cell proliferation, leukocyte fraction and CTA score. *P < 0.05, **P < 0.01, ***P < 0.001, n.s, not significant

Evaluating the seven steps of the immune cycle revealed considerable differences between high-risk and low-risk subtypes (Fig. 6A). The SubMAP algorithm predicted the likelihood of a response to CTLA-4 inhibition between the subgroups (Fig. 6B). Validation with four immunotherapy agents indicated that CTLA-4 treatment could be more effective in low-risk patients (Fig. 6C). The risk score was inversely related to the expression of immune inhibitors such as PD-1, PD-L1, HAVCR2, LAG-3, and CTLA-4 (Fig. 6D). Additionally, the low-risk subgroup was more likely to benefit from combination therapies involving PD-1 and CTLA-4 inhibitors (Fig. 6E).

These findings highlight the distinct immune characteristics between high-risk and low-risk glioblastoma subgroups, emphasizing the potential for personalized immunotherapeutic strategies based on risk stratification.

### High-risk patients are sensitive to topotecan

**Fig. 7:**
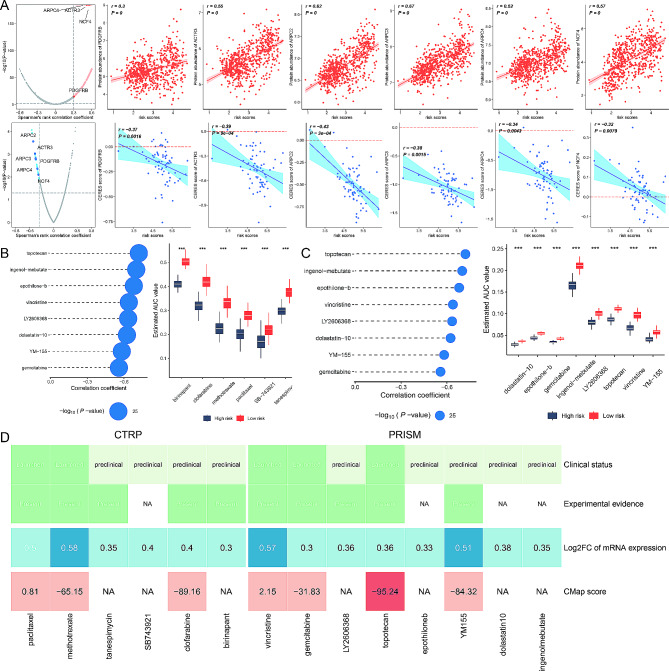
High-risk Patients are Sensitive to Topotecan. (**A**) Scatter plots showing the correlation between the risk score and the expression levels of druggable genes. (**B**) Bubble plot and box plots from the CTRP dataset, indicating the correlation coefficient and estimated AUC values for various compounds. (**C**) Bubble plot and box plots from the PRISM dataset, showing the correlation coefficient and estimated AUC values for compounds. (**D**) Comparative analysis from the CMap and PubMed literature review. CMap analysis identified topotecan as having gene expression patterns opposite to those in glioblastoma with a CMap score below − 95. Literature review provided experimental and clinical evidence supporting the efficacy of the identified compounds in glioblastoma treatment, with topotecan showing the most significant therapeutic potential

Genes that exhibit a strong positive correlation with the risk score might have therapeutic potential for high-risk patients. To identify druggable therapeutic targets for glioblastoma with poor prognoses, we calculated the correlation coefficient between the gene profiles and the risk score. Simultaneously, a correlation study between the CERES score and the risk score was performed. This analysis revealed six genes—*ARPC2*, *ARPC3*, *ARPC4*, *ACTR3*, *NCF4*, and *PDGFRB*—as potential therapeutic targets (Fig. 7A).

Using the CTRP and PRISM datasets, we created a predictive model for drug response. To find agents with increased drug sensitivity in high-risk patients, we conducted a comparative analysis of differential drug responses between high-risk and low-risk groups. This analysis identified compounds with lower estimated AUC values in the high-risk group. Additionally, a Spearman correlation analysis between AUC values and risk scores was performed, identifying compounds with a negative correlation coefficient (Spearman’s *r* < -0.30). This approach highlighted six compounds from the CTRP and eight from PRISM, all showing reduced AUC values and negative correlations with risk scores (Fig. 7B, C).

Despite these promising findings, increased drug sensitivity alone does not confirm the therapeutic efficacy of the 14 identified compounds for glioblastoma. To further evaluate their potential, a CMap analysis was conducted, identifying compounds with gene expression patterns opposite to those in glioblastoma. Topotecan emerged with a CMap score below − 95, indicating significant therapeutic potential. Additionally, a literature review on PubMed was performed to gather experimental and clinical evidence supporting the efficacy of these compounds in glioblastoma treatment, with cumulative results presented in Fig. 7D.

These analyses suggest that the compounds identified have promising therapeutic potential for glioblastoma, particularly for patients with high-risk scores. However, further experimental validation and clinical trials are essential to confirm their efficacy.

## Discussion

Glioblastoma is a malignant tumor characterized by high morbidity and mortality. Despite numerous studies aimed at improving the prognosis for glioblastoma patients, the median overall survival (OS) remains less than two years post-diagnosis. Currently, stage-based clinical approaches are limited in their ability to predict survival accurately. With the advent and advancement of precision medicine, there is an urgent need for a more accurate method to evaluate the prognosis of glioblastoma patients and guide treatment. Recent research on epigenetic biomarkers has significantly enhanced the precision of prognosis prediction and therapy measures for glioblastoma patients [24, 25]. Studies have shown that histone acetylation-related genes play a crucial role in tumor development and therapy. This study aims to construct a histone acetylation risk model based on five histone acetylation regulators, offering a novel strategy for identifying appropriate treatment methods for glioblastoma patients. While some researchers have developed models related to glioblastoma prognosis, no studies have established a histone acetylation model based on histone acetylation-related regulators to evaluate glioblastoma prognosis. According to our study, glioblastoma patients were divided into low- and high-risk groups. Low-risk patients had longer OS, increased immune infiltration, and were more sensitive to anti-CTLA4 immunotherapy, whereas the high-risk group responded better to chemotherapy, such as topotecan.

Furthermore, five histone acetylation regulators were selected to be considered as the significant glioblastoma prognosis genes, in which *HDAC1*, *HDAC3*, *HDAC4* and *HDAC7* belong to histone deacetylases differently, *HAT1* is a histone acetylase. In the study, we found that except for *HDAC4*, the other four regulators are positive with a risk score. It is indicated that *HDAC4* has a better prognosis, while, accordingly, *HAT1*, *HDAC1*, *HDAC3* and *HDAC7* may have a poor prognosis for glioblastoma. *HAT1*, Histone acetyltransferase 1, protein is a conserved enzyme that produces a marked effect in modifying histones via acetylating lysine residues [24], which is involved in chromatin assembly. Still, its function has not been explicitly elucidated. *Hat1* is the main *H4K5* and *H4K12* specific acetyltransferase in embryos, and its deletion results in changes in the transcription level of more than 2000 genes [26]. Based on genome-wide analysis, relevant studies have determined that *HAT1* is a type B histone acetyltransferase, a necessary gene upregulated in glioblastoma [27]. Under hypoxic conditions, the level of *HIF2A* protein in glioblastoma is regulated in a HAT1-dependent manner. The HAT1-HIF2A axis is vital for maintaining and reprogramming hypoxic cancer stem cells. Several researchers have also penetrated that HDAC is essential for maintaining the characteristics related to cancer stem cells (CSC) in malignant tumors [28]. Studies have demonstrated a correlation between histone deacetylase (HDAC) activity disorders and many oncological diseases [28–32]. For instance, Suberoylanilide Hydroxamic Acid (SAHA), an HDAC inhibitor (HDACI), causes checkpoint activation and induces tumor apoptosis in glioblastoma diseases [31]. HDAC1/2/6/Sp1 activation is bound up with poor prognosis in suffers with glioma [33]. *HDAC3* is also a histone deacetylase that maintains chromatin structure and genomic stability as an epigenetic regulator of gene expression [32]. The abnormally high expression of this enzyme in breast cancer, gastric cancer and acute lymphoblastic leukemia cells is associated with poor prognosis of these patients [34–36].

Similarly, *HDAC3* also takes effect in the chemoresistance of hypoxic glioblastoma. For example, Li et al. have shown that *HDAC3* inhibitor can prevent the presence of the drug resistance of glioblastoma to temozolomide [37]. Yixing Gao et al. demonstrate that *HDAC3* can regulate the expression of *MRP* through *MYCN*, strengthening the drug resistance of glioblastoma [38]. Based on the function of *HDAC3* in glioblastoma, it has been considered a potential therapy molecular to conquer the drug resistance of glioblastoma. *HDAC7* is a class II histone deacetylase that works in the production of the vascular system. Related studies have found that silencing *HDAC7* can inhibit the angiogenesis of endothelial cells [39–41]. Angiogenesis is a marker of tumor occurrence and development, including glioblastoma. Xiao et al. [25] found that *HDAC7* is able to inhibit STAT3 activity via specific interplay with Tip60. Silencing of *HDAC7* can induce the activation of STAT3 in endothelial cells, preventing this cell migration and differentiation. Animal studies on orthotopic xenograft tumors have shown that causing inhibition of *HDAC7* can lead to a 3-fold reduction in tumor volume [42]. Glioblastoma is addicted to angiogenesis. Inhibiting *HDAC7* can inhibit angiogenesis and cut off oxygen and nutrients from cancer cells. Unfortunately, up to now, there are no specific inhibitors of *HDAC7*, which makes its targeting extremely difficult. Overall, these results highlight *HDAC7* may be as a promising target in glioblastoma. Based on the above results, it is indicated that five histone acetylation regulators selected make a difference in glioblastoma development and are all under consideration as a potential therapeutic factor for glioblastoma therapy.

Recent studies have identified several prognostic gene signatures in glioblastoma that highlight the complex interplay between immune evasion and cancer prognosis. Notably, genes such as *CD276* (B7-H3), *GATA3*, and *LGALS3* (galectin-3) have been shown to play significant roles in glioblastoma prognosis [43]. These genes are involved in various immune regulatory mechanisms that enable tumor cells to evade immune surveillance, contributing to disease progression and poor clinical outcomes. Additionally, there is evidence suggesting that the status of Th2 cells and the expression of PD-L1/PD-1 axis genes are closely linked to glioblastoma prognosis [44]. Specifically, a lower expression of PD-L1 and PD-1 axis genes, coupled with an altered Th2 cell status, has been associated with better prognostic outcomes. These findings underscore the critical role of immune checkpoints and T cell polarization in shaping the tumor microenvironment and influencing patient survival. Our study builds on these insights by demonstrating that histone acetylation, a key epigenetic modification, significantly impacts immune cell infiltration and activity in glioblastoma. The positive correlation between the HA-score and M2 macrophages, along with the negative correlation with CD8^+^ T cells, suggests that histone acetylation may contribute to an immunosuppressive microenvironment. This, in turn, may facilitate immune evasion and adversely affect patient prognosis. By integrating our findings with these previous studies, we provide a comprehensive view of the molecular mechanisms underpinning immune evasion in glioblastoma. Our histone acetylation risk model not only offers prognostic value but also highlights potential therapeutic targets for modulating the immune landscape in glioblastoma.

In addition to immune evasion, the cancer microenvironment in glioblastoma is significantly influenced by processes such as epithelial-mesenchymal transition (EMT) and the properties of cancer stem cells (CSCs). EMT is a biological process where epithelial cells acquire mesenchymal characteristics, enhancing their migratory and invasive capabilities. This transition is crucial for tumor progression and metastasis, and it is also associated with resistance to apoptosis and therapy [45]. In glioblastoma, EMT contributes to the aggressive nature of the disease. Key EMT markers such as E-cadherin and N-cadherin are indicative of this transition. EMT not only promotes invasiveness but also modulates the immune microenvironment, facilitating immune escape. Tumor cells undergoing EMT can evade immune detection by altering the expression of immune checkpoint molecules and secreting immunosuppressive cytokines. Furthermore, CSCs play a pivotal role in glioblastoma progression and recurrence. CSCs possess the ability to self-renew and differentiate into various cell types within the tumor, contributing to tumor heterogeneity and therapeutic resistance. CSCs are also known to interact with the immune microenvironment, creating a niche that supports their survival and proliferation. These interactions involve the secretion of factors that suppress immune cell activity and promote an immunosuppressive microenvironment. Our study highlights the role of histone acetylation in modulating the immune microenvironment in glioblastoma. The positive correlation between the HA-score and M2 macrophages, along with the negative correlation with CD8^+^ T cells, suggests that histone acetylation may influence EMT and CSC properties indirectly by shaping an immunosuppressive niche. This comprehensive view underscores the multifaceted nature of glioblastoma’s microenvironment, where immune escape, EMT, and CSC properties converge to drive tumor progression and resistance.

While our model shows promising results, there are several limitations to consider. First, the study’s retrospective nature may introduce biases, and prospective validation in clinical trials is necessary to confirm these findings. Additionally, the model’s applicability across diverse populations and different glioblastoma subtypes needs further investigation. Future research should focus on integrating other epigenetic markers and molecular profiles to refine the model further. Exploring the mechanistic pathways linking histone acetylation to immune modulation could also provide deeper insights into glioblastoma biology and uncover new therapeutic targets.

## Conclusions

In summary, our histone acetylation risk model offers a novel and effective tool for predicting glioblastoma prognosis and guiding therapy selection. By accurately identifying low-risk and high-risk patients, this model paves the way for personalized treatment strategies that could improve patient outcomes. The integration of immune profiling and drug sensitivity analysis further enhances the model’s clinical utility, highlighting its potential to revolutionize glioblastoma management.

Our findings underscore the critical role of histone acetylation in glioblastoma progression and treatment response. By advancing our understanding of these molecular mechanisms, we can develop more targeted and effective therapies, ultimately improving survival rates and quality of life for glioblastoma patients.

### Electronic supplementary material

Below is the link to the electronic supplementary material.

Supplementary Material 1

Supplementary Material 2

Supplementary Material 3

## Data Availability

The datasets used and/or analysed during the current study are available from the corresponding author on reasonable request.
