# Supplementary material for: Histone acetylation risk model predicts prognosis and guides therapy selection in glioblastoma: implications for chemotherapy and anti-CTLA-4 immunotherapy
Source: BMC Immunol. 2024 Jul 27;25:51. doi: 10.1186/s12865-024-00639-7 (PMC11282667; doi:10.1186/s12865-024-00639-7)
Supplement: Supplementary file 1 — Supplementary Material 1 [file 12865_2024_639_MOESM1_ESM.pdf]

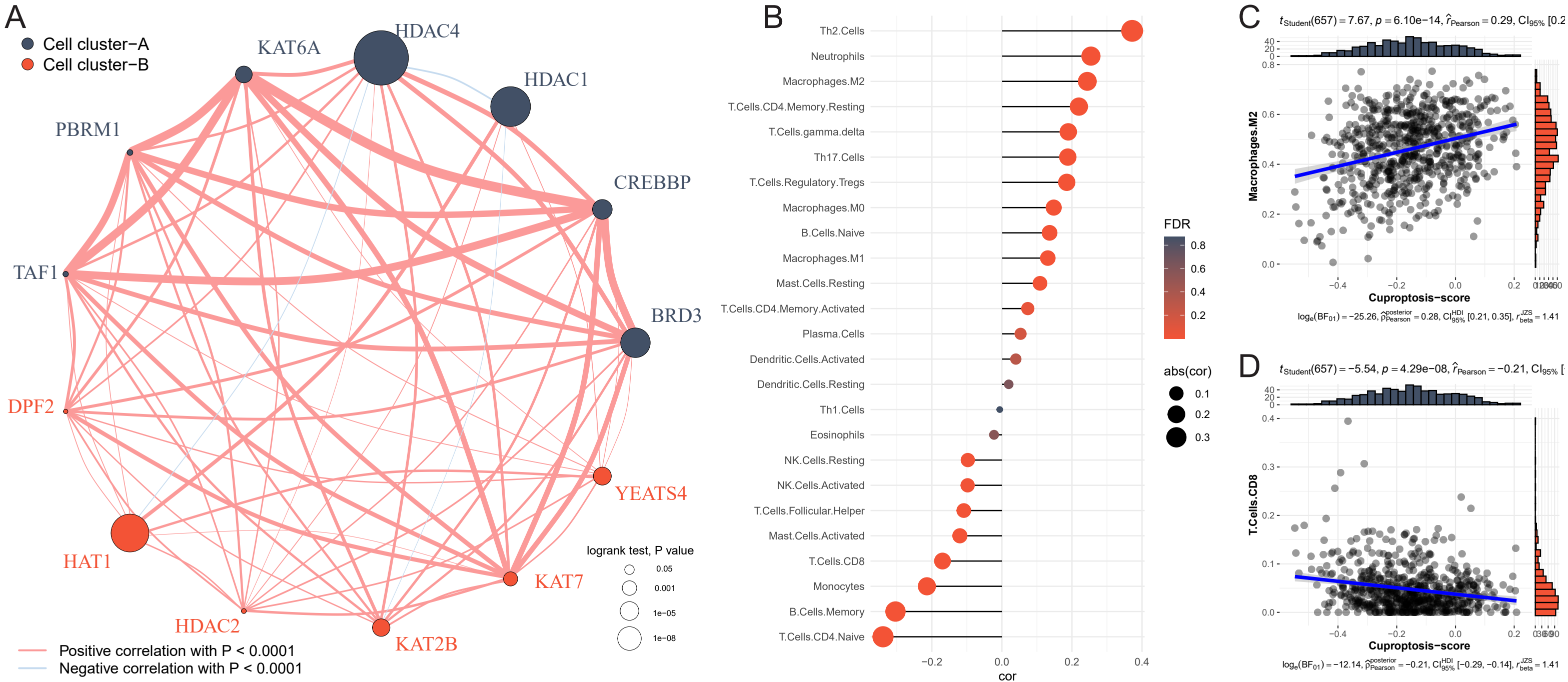

Figure S1. HA-score correlates immune cell infiltration. (A) Correlation network of the 15 differential histone acetylation regulators categorized into two clusters. (B) Dot plot showing the correlation between the HA-score and immune cell infiltration. (C, D) Scatter plots illustrating the correlation between the HA-score and the presence of M2 macrophages and CD8+ T cells, respectively.
