# Supplementary material for: Histone acetylation risk model predicts prognosis and guides therapy selection in glioblastoma: implications for chemotherapy and anti-CTLA-4 immunotherapy
Source: BMC Immunol. 2024 Jul 27;25:51. doi: 10.1186/s12865-024-00639-7 (PMC11282667; doi:10.1186/s12865-024-00639-7)
Supplement: Supplementary file 2 — Supplementary Material 2 [file 12865_2024_639_MOESM2_ESM.pdf]

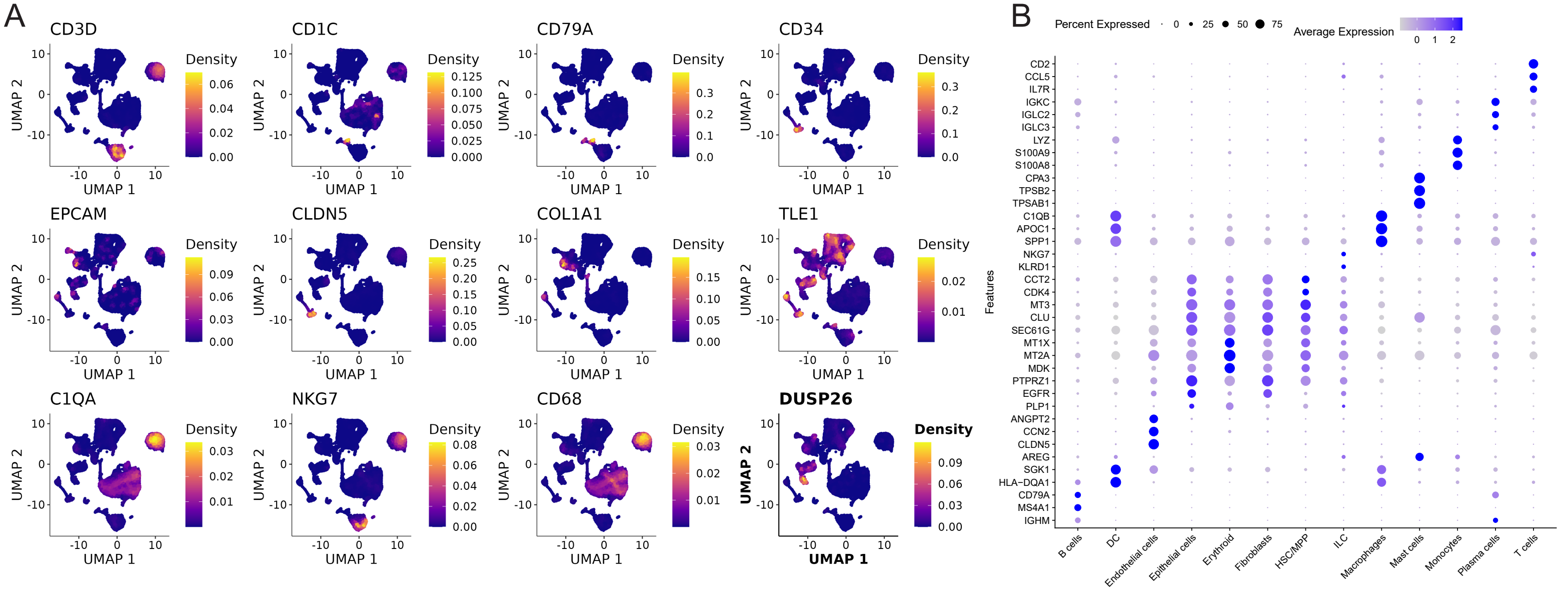

Figure S2. Representative markers across cell type. (A) UMAP plots labeling the representative markers for each cell type. (B) Dot plot highlighting the top differential genes across the different cell types.
