# Supplementary material for: Histone acetylation risk model predicts prognosis and guides therapy selection in glioblastoma: implications for chemotherapy and anti-CTLA-4 immunotherapy
Source: BMC Immunol. 2024 Jul 27;25:51. doi: 10.1186/s12865-024-00639-7 (PMC11282667; doi:10.1186/s12865-024-00639-7)
Supplement: Supplementary file 3 — Supplementary Material 3 [file 12865_2024_639_MOESM3_ESM.pdf]

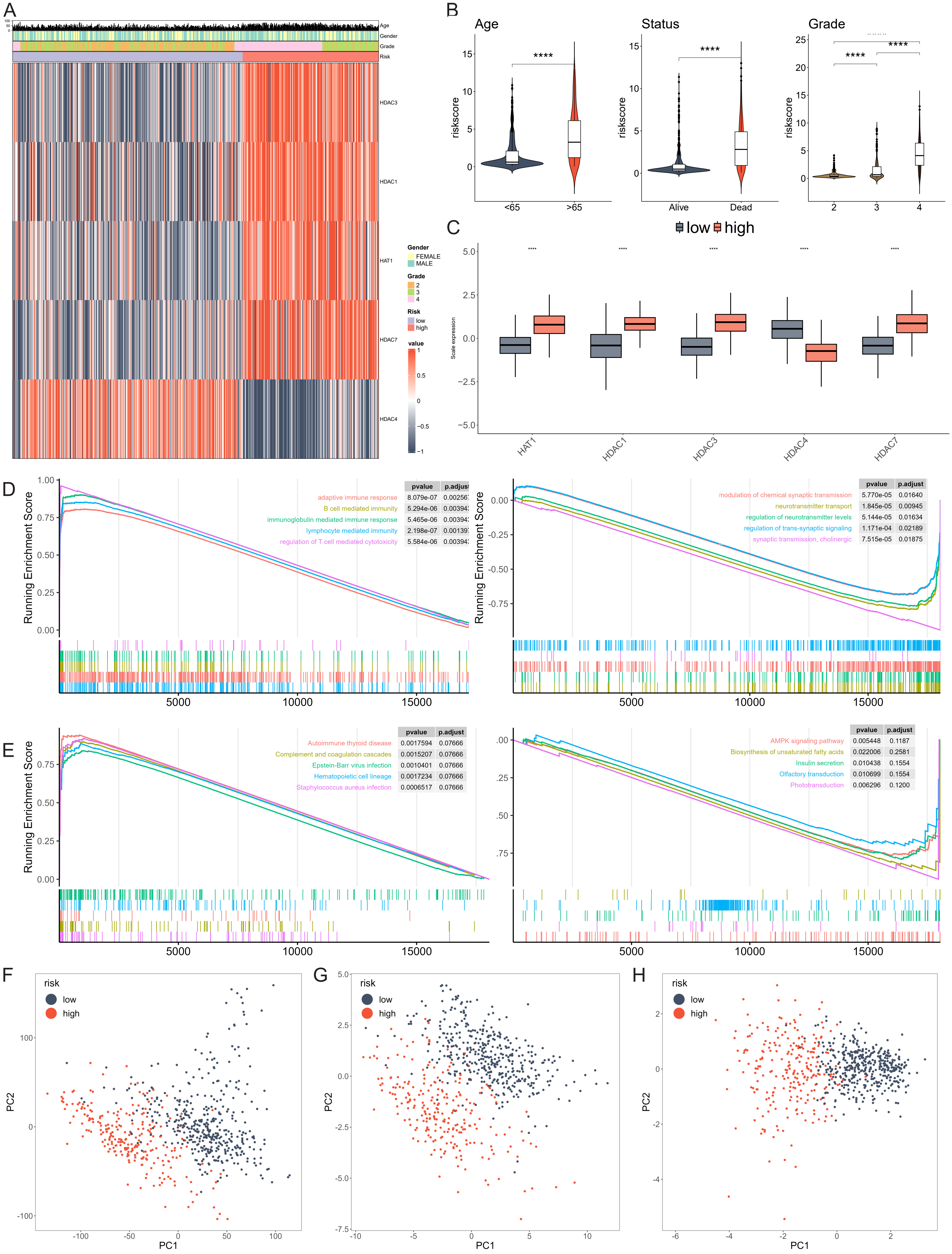

Figure S3. Significance of HA-model to clinical indexes and functional variations. (A) Heatmap showing the distribution patterns of five histone acetylation regulators and various clinicopathological variables among glioblastoma patients. (B) Violin plots demonstrating the HA-model's predictive value for age, OS status, and clinicopathological grade. (C) Box plots showing the correlation between the expression levels of five histone acetylation regulators and the risk score. (D, E) GSEA identifying significant pathways in the high-risk subgroup. (F) PCA on the entire gene set distinguishing high-risk and low-risk subgroups. (G) PCA based on histone acetylation regulators showing clear separation between the two subgroups. (H) PCA using the five selected histone acetylation regulators from the HA-model, successfully differentiating the high-risk and low-risk subgroups.
